# Meta-analysis of vitamin D and lung function in patients with asthma

**DOI:** 10.1186/s12931-019-1072-4

**Published:** 2019-10-08

**Authors:** Jian Liu, Yong-Quan Dong, Jie Yin, Jian Yao, Jie Shen, Guo-Jie Sheng, Kun Li, Hai-Feng Lv, Xing Fang, Wei-Fang Wu

**Affiliations:** 1 Department of Intensive Care Unit, the First Affiliated Hospital, College of Medicine, Zhejiang University, Hangzhou, Zhejiang Province China; 2 Department of Respiratory Disease, Yinzhou No. 2 Hospital, Ningbo, Zhejiang Province China; 3 Department of Emergency, People’s Hospital of Jinyun County, LiuShui, Zhejiang Province China; 4 Department of Medical Oncology, The First Affiliated Hospital, College of Medicine, Zhejiang University, Hangzhou, Zhejiang Province China

**Keywords:** Vitamin D, Asthma, Lung function

## Abstract

**Background:**

There is growing literature suggesting a link between vitamin D and asthma lung function, but the results from systematic reviews are conflicting. We conducted this meta-analysis to investigate the relation between serum vitamin D and lung function in asthma patients.

**Methods:**

Major databases, including OVID, MEDLINE, Web of Science and PUBMED, were searched until 10th October 2018. All published observational studies related to vitamin D and asthma were extracted. All meta-analyses were performed using Review Manager 5.3.5.

**Results:**

This quantitative synthesis found that asthma patients with low vitamin D levels had lower forced expiratory volume In 1 s (FEV1) (mean difference (MD) = − 0.1, 95% CI = − 0.11 to − 0.08,*p* < 0.01;I2 = 49%, *p* = 0.12) and FEV1% (MD = − 10.02, 95% CI = − 11 to − 9.04, p < 0.01; I2 = 0%, *p* = 0.82) than those with sufficient vitamin D levels. A positive relation was found between vitamin D and FEV1 (r = 0.12, 95% CI = 0.04 to 0.2, *p* = 0.003; I2 = 59%,*p* = 0.01), FEV1% (r = 0.19, 95% CI = 0.13 to 0.26, *p* < 0.001; I2 = 42%, *p* = 0.11), forced vital capacity (FVC) (r = 0.17, 95% CI = 0.00 to 0.34, *p* = 0.05; I2 = 60%, *p* = 0.04), FEV1/FVC (r = 0.4, 95% CI = 0.3 to 0.51, *p* < 0.001; I2 = 48%, *p* = 0.07), and the asthma control test (ACT) (r = 0.33, 95% CI = 0.2 to 0.47, p < 0.001; I2 = 0%, *p* = 0.7). Subgroup analysis indicated that the positive correlation between vitamin D and lung function remained significant in both children and adults.

**Conclusions:**

Our meta-analysis suggested that serum vitamin D levels may be positively correlated with lung function in asthma patients. Future comprehensive studies are required to confirm these relations and to elucidate potential mechanisms.

## Background

Asthma is a common inflammatory disorder of the air passages that involves many cellular elements, such as mast cells, eosinophils and T helper (Th) lymphocytes. Asthma is a major public health concern worldwide because of the increasing prevalence of the condition, along with its negative influence in the community with regard to extensive health care costs and deteriorating quality of life [1]. The occurrence and development of asthma is influenced by various factors, incorporating genetic, environmental, and ethnic factors, as well as socioeconomic status [2]. A subgroup of asthmatics was shown to manifest a decreased response to standard therapy and experience poorer lung function and more frequent exacerbation. To improve clinical outcomes of patients with asthma and quality of life, methods to slow asthma lung function exacerbations are urgently needed.

The results from studies of vitamin D and the clinical prognosis of asthma patients have led to conflicting messages. A few observational studies suggested that lower 25(OH)D levels were associated with worse glucocorticoid responsiveness, greater exacerbation frequency, worse lung function and substantially more severe asthma symptoms [3–5]. However, Kang, Q. et al. [6] enrolled 96 children with asthma and found that vitamin D levels were not associated with FEV1 (forced expiratory volume in one second), FVC (forced vital capacity) and FEV1/FVC levels (*p* > 0.05). Reviews and meta-analysis evaluating the current evidence of the association between serum vitamin D and asthma lung function have also been published; two reported no protective effects of vitamin D supplementation on asthma lung function [7, 8], two reported positive effects [9, 10], and two did not conduct a meta-analysis of the outcome of pulmonary function [11, 12]. The conclusions are mixed, and furthermore the study populations included only children. However, more research has been published on this subject in recent years. Hence, we conducted a systematic meta-analysis of observational studies with substantially more evidence to specifically investigate the relation between serum 25(OH)D and asthma lung function in both adults and children.

## Methods

The Meta-analysis of Observational Studies in Epidemiology (MOOSE) [13] and the Preferred Reporting Items for Systematic Reviews and Meta-Analyses (PRISMA) [14] were applied in this study.

### Eligibility criteria

Eligible clinical studies were defined based on the following criteria: (1) study design was observational; (2) participants were humans of all ages; (3) asthma was diagnosed according to the Global Initiative for Asthma (GINA) or relevant guidelines; (4) content was related to vitamin D and asthma; (5) outcome measures included lung function, such as FEV1, FEV1%, FVC, and asthma symptom scores such as the Asthma Control Test (ACT); and (6) language was restricted to English only.

### Exclusion criteria

(1) Randomised controlled trials (RCTs) evaluating the relation between vitamin D supplementation and asthma, as this was not the goal of the review; (2) studies assessing maternal serum levels of vitamin D and the incidence of asthma in children.

### Data sources and study selection

An electronic search was conducted in the following databases: OVID, MEDLINE, Web of Science and PUBMED, for studies published up to 10th October 2018 using the key words asthma, vitamin D, and lung function. The search strategy was developed by two investigators in duplicate and independently according to standardised and pilot-tested selection criteria. Review articles and reference lists of observational publications were manually searched for any possible supplementary references. Any dispute was resolved by mutual consensus with a third investigator.

### Data extraction and quality assessment

Independently, the same reviewers extracted and recorded necessary information from each enrolled study using a standard form recommended by Cochrane [15], which included publication year, study design, authors, country, participants and population, demographic characteristics and measurements. In cases of missing data, we contacted the corresponding authors via email to ask for the full original data. Once extraction was completed, the data were reviewed to identify duplicate studies and duplicate reporting of populations; only the most comprehensive studies were retained.

The Newcastle–Ottawa Scale (NOS) was applied to assess the quality of the observational studies (case–control and cohort studies) [16]. Two independent assessors conducted the quality assessment, and any disagreement was settled by reaching a consensus or consulting a third researcher.

### Statistical methods

An estimate of the pooled correlation coefficient (r) between vitamin D and asthma lung function was calculated by combining the standard errors (SEs), and Fisher’s z transformation was calculated by the following formulas. We calculated SEs via formula 1 and formula 2. Each correlation coefficient was transformed by Fisher’s z formula (Formula 3). The pooled results were calculated using the generic inverse variance method, after which all the values were converted back to the original correlation coefficient metric (Formula 4). (Formulas 1–4 are shown in Additional file 2: Figure S1) [17, 18]. For studies reporting their outcomes as continuous data, we counted the mean difference when studies used the same measurement and the standardised mean difference when they used different measurements. We tested the results for homogeneity by using the I2 statistics (I2≧75% for evident heterogeneity) and Q (*p* > 0.10 in the Chi-square test for low heterogeneity) [19]. The obtained data were pooled with the DerSimonian & Laird random-effects models to acclimatise variety [20]. Otherwise, a fixed-effects model was used when there was no obvious heterogeneity [21]. A *P* value less than 0.05 was considered statistically significant. Funnel plots were applied to explore the possibility of publication bias. For studies providing r2 values, the r values were calculated using the r2 values in the paper and by measuring the graphical representation to confirm the sign. For statistical convenience, the quartile was converted to the mean and standard deviation and the unit of measurement for serum vitamin D levels was unified to ng/ml. Sensitivity analysis was executed by omitting each study in turn to evaluate the reliability and validity of the pooled results. Review Manager (Version 5.3.5, The Nordic Cochrane Centre, The Cochrane Collaboration, Copenhagen, 2014) was applied for data acquisition and analysis.

## Results

### Search results

**Fig. 1:**
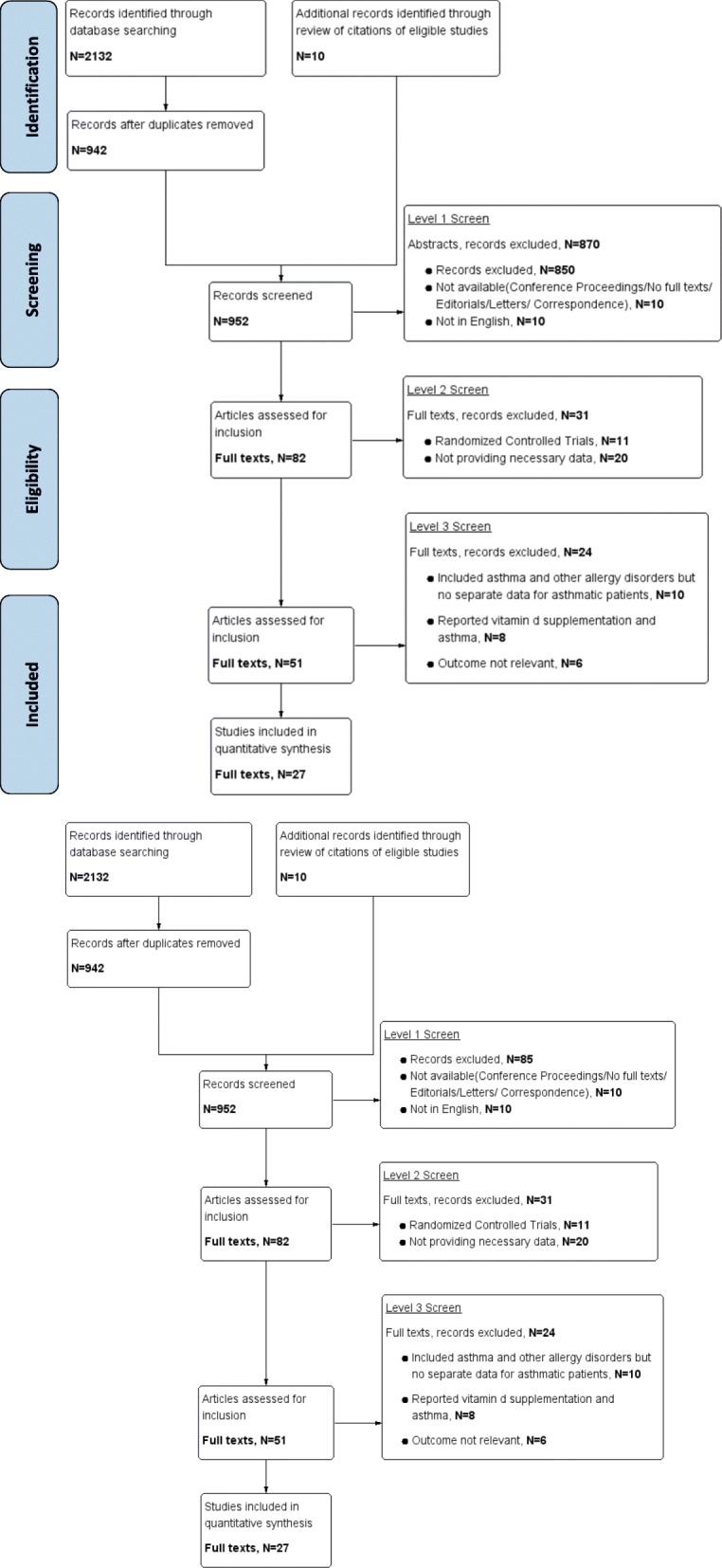
Flow diagram of study selection process

The flow diagram for study selection is displayed in Fig. 1. The electronic database search identified a total of 2142 citations. After duplicate publications were removed, 942 studies were included. After evaluating the titles and abstracts at level 1 screening, 82 records were included. Assessment of the full text at level 2 screening removed another 24 articles. In total, 27 studies were ultimately included. A majority of the enrolled studies were of moderate to high quality based on the NOS scores.

### Study characteristics

Table 1 and Table 2 describe the characteristics of the included studies. The 27 studies were published in English between 2010 and 2018. There were 19 cross-sectional studies, six case–control studies and 2 cohort studies. Eighteen articles evaluated children, and the remaining 9 articles involved adults. Included studies were from both developing and developed countries: six studies were conducted in the USA, four in Turkey, three in China, two in Italy, two in the UK, two in Germany, and two in Thailand, while the other 6 were conducted in Iran, Boston, India, Jordan, Saudi Arabia and Macedonia. The extracted data about outcome measurements were as follows: the correlations between vitamin D and FEV1% (10 studies), FEV1 (13 studies), FEV1/FVC (12 studies), FVC% (2 studies), FVC (8 studies), ACT (asthma control test) scores (6 studies). Our analysis also included three groups to compare the mean difference in FEV1% (4 studies), FEV1 (8 studies), and FEV1/FVC (6 studies) between vitamin D deficiency and insufficiency patients and vitamin D sufficiency patients.

**Table 1:** Characteristics of included studies about the relation between vitamin d and lung function in asthma patients

Author (year)	Country	Research	Partients	Ages	Measurement	VitaminD level (ng/ml)	rFEV1%	rFEV1	rFVC	FVC %	rFEV1/FVC	ACT
Searing,D.A2010 [22]	USA	cross-sectional	100	7 (4–10)	CLIA	median value:31	0.34	NG	NG	0.12	0.3	NG
Sutherland,E.R.2010 [23]	USA	cross-sectional	54	38.3 ± 11.2	LC-MS/MS	28.1 ± 10.2	NG	0.8	NG	NG	NG	NG
Alyasin, S.2011 [24]	Iran	cross-sectional	50	9.31 ± 2.67	RIA	49.29 ± 21.44	NG	0.564	NG	NG	0.561	NG
Chinellato, I.2011(a) [25]	Italy	cross-sectional	75	9.6 ± 1.7	CLIA	NG	0.16	NG	0.25	NG	−0.15	0.28
Chinellato, I.2011(b) [26]	Italy	cross-sectional	45	10 (9–11)	RIA	19.7 ± 5.778	NG	0.32	0.34	NG	NG	NG
Li,F2011 [27]	Chinese	cross-sectional	435	42.6 ± 1.6	ELISA	Median 30.53	0.12	NG	NG	NG	NG	NG
Gupta, A. 2012 [28]	UK	cross-sectional	86	11.7	HPLC	34.08 ± 19.8	NG	0.43	0.32	NG	NG	0.6
Korn,S.2013 [29]	Germany	cross-sectional	280	45.0 ± 13.8	RIA	25.6 ± 11.8	0.235	NG	NG	NG	NG	NG
Krobtrakulchai,W.2013 [30]	Thailand	cross-sectional	125	10.8 ± 3.0	ECLIA	27.8 ± 8.6	NG	0.09	−0.007	NG	0.124	NG
Montero-Arias, F.2013 [31]	USA	cross-sectional	121	48.1 ± 15.7	ELISA	NG	NG	0.173	NG	NG	NG	NG
Awasthi,S.2014 [32]	India	case–control	20	5–15 y	ELISA	20.84 ± 7.99	0.853	NG	NG	NG	NG	NG
Columbo, M.2014 [33]	USA	cross-sectional	28	72.6 ± 5.8	NG	24.3 ± 9.2	0.34	NG	NG	NG	0.11	NG
Dogru, M.2014 [34]	Turkey	case-control	120	4.4 ± 1.2 years	LC-MS	21.49 ± 7.74	NG	NG	NG	NG	NG	NG
Samrah, S.2014 [35]	Jordan	case–control	68	41 ± 13.7	HPLC	8.3 ± 3.2	NG	NG	NG	NG	NG	0.3
Aldubi, H. M.2015 [36]	Saudi	cross-sectional	45	9.2 ± 1.1	ECLIA	11.1 ± 5.75	NG	NG	NG	NG	NG	0.956
Tamasauskiene,L.2015 [37]	UK	case-control	85	46.41 ± 1.54	ELISA	14.36 ± 0.57	NG	−0.06	−0.01	NG	0.72	NG
Boonpiyathad,T.2016 [38]	Tailand	cross-sectional	47	63.48 ± 11.79	HPLC	23.84 ± 8.89	NG	NG	NG	NG	NG	0.3
Havan, M.2017 [39]	Turkey	cross-sectional	38	10.28 ± 2.70	CLIA	14.44 ± 6.203	NG	0.122	NG	NG	0.633	NG
Havan, M.2017 [39]	Turkey	cross-sectional	20	10.28 ± 2.70	CLIA	14.44 ± 6.203	NG	0.136	NG	NG	0.136	NG
Havan, M.2017 [39]	Turkey	cross-sectional	14	10.28 ± 2.70	CLIA	14.44 ± 6.203	NG	0.167	NG	NG	0.549	NG
Janeva-Jovanovska,E.2017 [40]	Macedonia	cross-sectional	30	NG	ELISA	15.260 ± 5.808	NG	−0.1005	NG	NG	NG	NG
Ozdogan, S. 2017 [41]	Turkey	cross-sectional	71	11.97 ± 1.93	LC-MS/MS	11.8 ± 10.3	− 0.184	−0.17	− 0.05	0.01	− 0.22	NG
Bai,Y.J2018 [42]	China	case-control	117	8.52 ± 2.37	CLIA	6.9 ± 1.77	0.79	NG	0.77	NG	0.7	NG
Batmaz,S.B.2018 [43]	Turkey	cohort	30	11.74 ± 2.4	HPLC	25.74 ± 9.06	0.483	NG	NG	NG	NG	0.498
Kang, Q.2018 [6]	China	case-control	96	6.56 ± 1.38	ELISA	18.89 ± 3.63	NG	−0.568	0.601	NG	0.345	NG
Reinehr, T.2018 [44]	Germany	cross-sectional	36	9.3 ± 1.7	CLIA	20.6 ± 9.1	0.001	NG	NG	NG	NG	NG

*NG* Not given, *RIA* Radio-immunoassay, *CLIA* Chemiluminescent immunoassay, *HPLC* High-performance liquid chromatography, *ECLIA* Electroluminescence immunoassay, *ELISA* Enzyme-linked immunosorbent assay, *LC-MS/MS* Liquid chromatography tandem mass spectrometry, *USA* United States, *UK* United Kingdom

**Table 2:** Characteristics of the included studies about vitamin d levels in asthma patients

Author (year)	Country	Research type	Measurement	Age	Extracted data
Brehm,J.M.2010 [45]	USA	cross-sectional	RIA	8.9 (7.2–10.6)	FEV1,FEV1/FVC
Alyasin,S.2011 [24]	Iran	cross-sectional	RIA	9.31 ± 2.67	FEV1/FVC
Li,F.2011 [27]	Chinese	cross-sectional	ELISA	42.6 ± 1.6	FEV1,FEV1/FVC,FEV1%
Brehm,John M.2012 [46]	USA	cross-sectional	HPLC	10.1 ± 2.6	FEV1,FEV1/FVC
Korn,S.2013 [29]	Germany	cross-sectional	RIA	45.0 ± 13.8	FEV1,FEV1%
Ozdogan, S.2017 [41]	Turkey	cross-sectional	LC-MS/MS	11.97 ± 1.93	FEV1,FEV1/FVC,FEV1%
Montero-Arias,F.2013 [31]	Costa Rica	cross-sectional	EIA	48.1 ± 15.7	FEV1/FVC,FEV1%
Wu,A.C.2012 [47]	Boston	cohort	RIA	8.94 ± 2.12	FEV1
					FEV1
					FEV1

Furthermore, the categorisation thresholds and definitions of 25(OH)D deficiency, insufficiency and sufficiency varied across the studies. The most frequently used categorical levels to describe serum 25(OH)D deficiency, insufficiency and sufficiency were < 20 ng/ml (50 nmol/l), 20–29.9 ng/ml (50–74.9 nmol/l) and ≥ 30 ng/ml (≥75 nmol/l), respectively.

### Different lung function in asthma patients with different vitamin D values

**Fig. 2:**
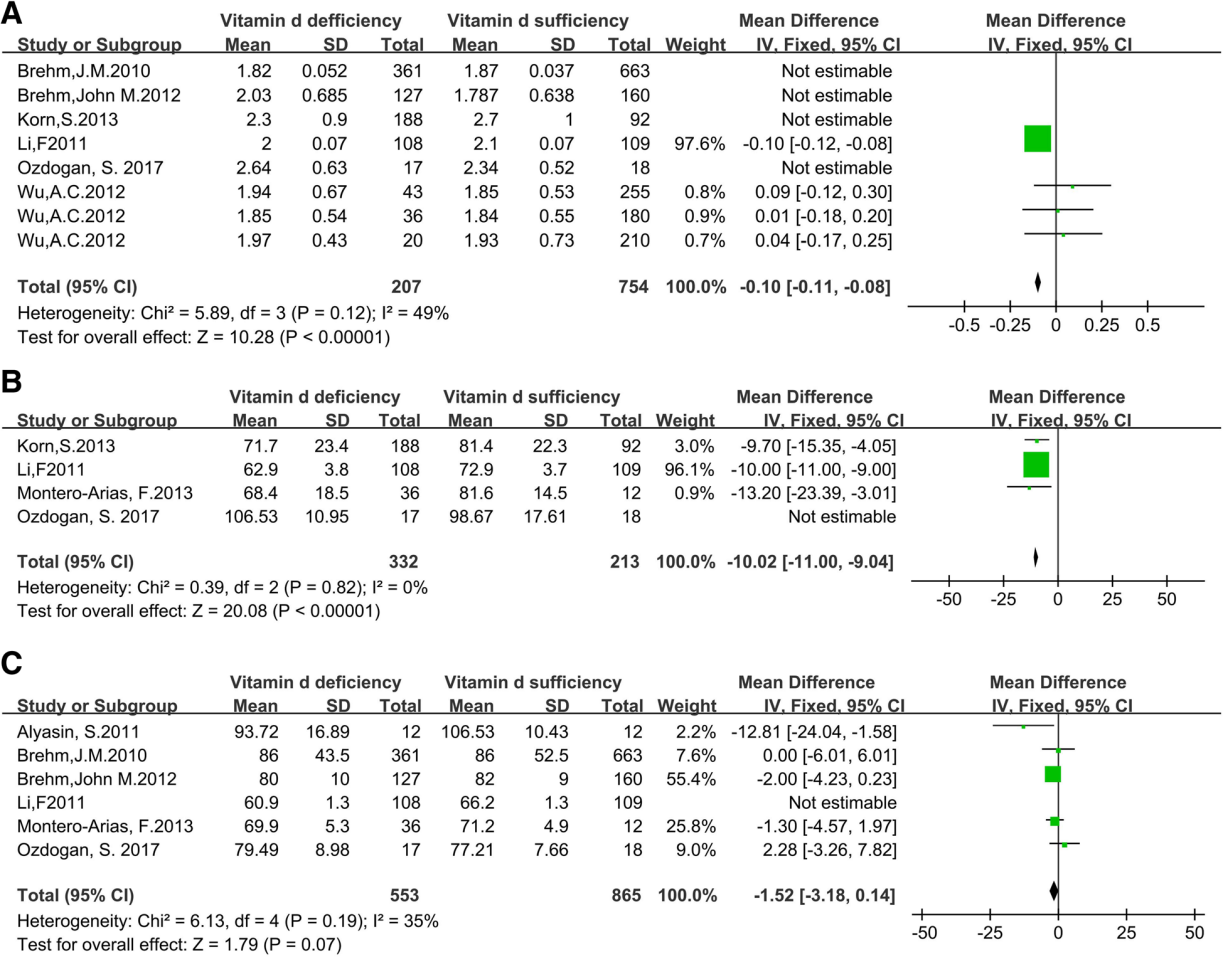
Forest plots analyzing lung function of asthma patients with different vitamin d levels **a** FEV1; **b** FEV1%; **c** FEV1/FVC

This quantitative synthesis found that asthma patients with low vitamin D levels had lower FEV1 (MD = − 0.05, 95% CI = − 0.06 to − 0.05, *p* < 0.01;I2 = 87%, *p* < 0.01), FEV1% (MD = − 7.11, 95% CI = − 13.23 to − 0.98, *p* = 0.02; I2 = 78%, *p* < 0.01) and FEV1/FVC (MD = − 5.14, 95% CI = − 5.48 to − 4.80, *p* < 0.01; I2 = 80%, *p* < 0.01) than those with sufficient vitamin D levels. Sensitivity analysis excluded four outliers [29, 45, 46] from the FEV1 group, one outlier [48] from the FEV1% group and one outlier [27] from the FEV1/FVC group. The final results were as follows: FEV1 (MD = − 0.1, 95% CI = − 0.11 to − 0.08,*p* < 0.01;I2 = 49%, *p*= 0.12), FEV1% (MD = − 10.02, 95% CI = − 11 to − 9.04, *p* < 0.01; I2 = 0%, *p* = 0.82) and FEV1/FVC (MD = − 1.52, 95% CI = − 3.18 to 0.14, *p* = 0.07; I2 = 35%, *p* = 0.19) (Fig. 2a-c).

### Relation between vitamin D and lung function in asthma patients

**Fig. 3:**
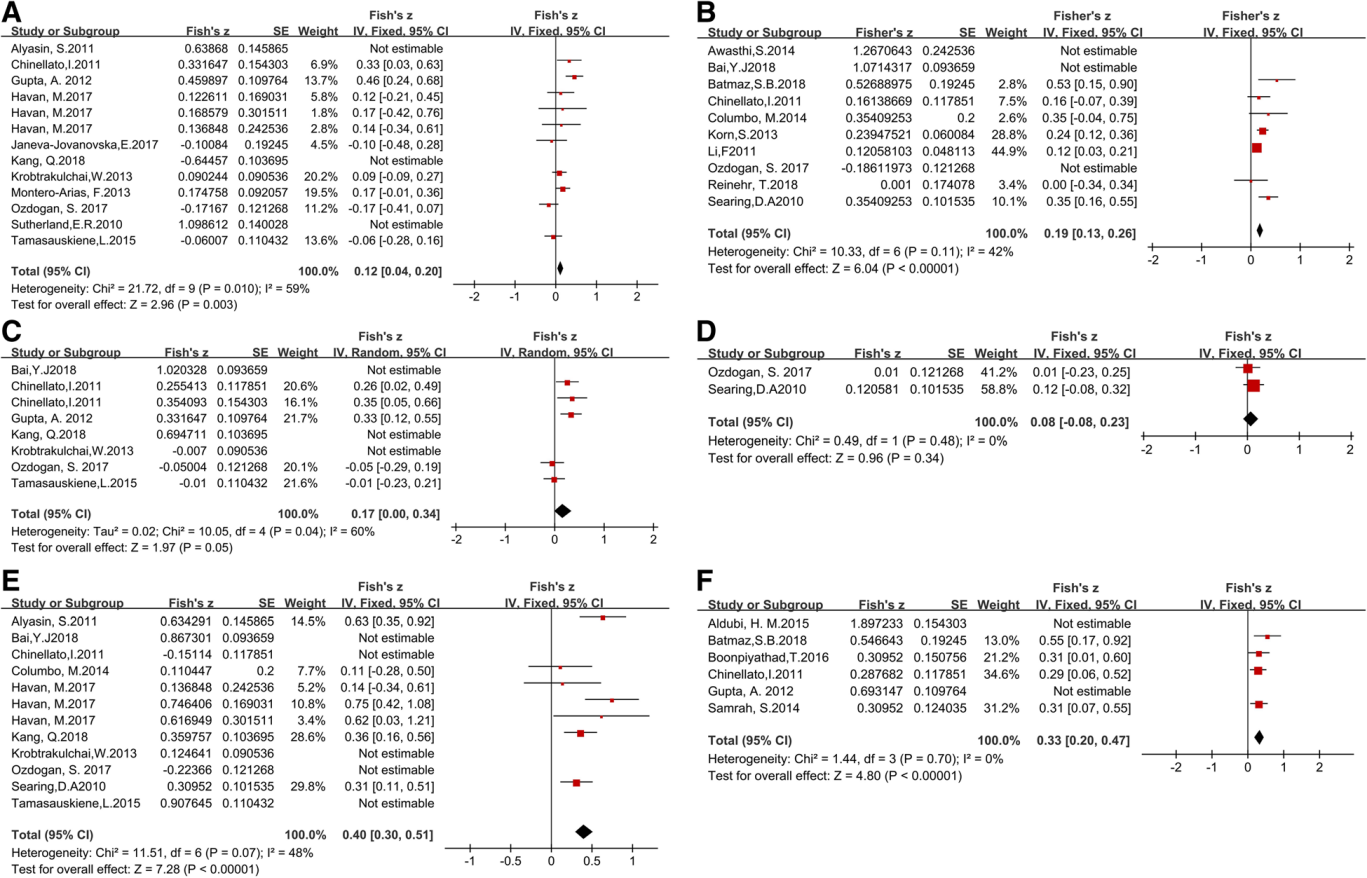
Forest plots of the relation between vitamin d and **a** FEV1; **b** FEV1%; **c** FVC%; **d** FVC; **e** FEV1/FVC; **f** ACT

Thirteen studies described the relationship between vitamin D and FEV1. A total of 290 adults and 545 children were included. The mean vitamin D concentrations were 21.5 ± 11.40 ng/ml in adults and 21.11 ± 14.50 ng/ml in children. The pooled r was 0.12 (95% CI 0.05–0.19, *p* < 0.001) and was highly heterogeneous I2 = 91% (p < 0.001). After sensitivity analysis, removing Alyasin, S. et al., Kang, Q.2018. et al. and Sutherland, E. R et al. [6, 23, 24], (the three outliers), this heterogeneity was significantly reduced with little impact on the outcome (r = 0.12, 95%CI = 0.04 to 0.2, *p* = 0.003; I2 = 59%, *p* = 0.01) (Fig. 3a).

Ten studies was selected to explore the association between vitamin D and FEV1% (r = 0.27, 95% CI = 0.22 to 0.33, *p* < 0.001; I2 = 93%, *P* < 0.001). Three studies [32, 42, 48] were regarded as homogeneous after a sensitivity analysis and were eliminated from the pooled result (r = 0.19, 95% CI = 0.13 to 0.26, p < 0.001; I2 = 42%, *P* = 0.11) (Fig. 3b).

Eight studies explored vitamin D and FVC with the pooled r = 0.33 (95% CI = 0.04–0.61, *p* = 0.03) and heterogeneity I2 = 93% (*p* < 0.001). Three outliers [6, 30, 42] were removed, resulting in a pooled r = 0.17 (95% CI = 0.00 to 0.34, *p* = 0.05), I2 = 60% (*p* = 0.04) (Fig. 3c). The pooled correlation between vitamin D and FVC% was r = 0.08 (95% CI = − 0.08 to 0.23, *p* = 0.34) with heterogeneity I2 = 0% (*p* = 0.48), which did not reach statistical significance (Fig. 3d).

The pooled result of FEV1/FVC with vitamin D was r = 0.38, (95% CI = 0.31 to 0.45, *p* < 0.001; I2 = 90%, p < 0.001). After the sensitivity analysis removed five outliers [25, 30, 37, 42, 48], we obtained the result of r = 0.4, (95% CI = 0.3 to 0.51, p < 0.001; I2 = 48%, *p* = 0.07) (Fig. 3e).

The pooled r for 6 studies with ACT scores was 0.62 (95% CI = 0.51 to 0.73, p < 0.001), with I2 = 94% (p < 0.001). Two studies providing discrete values [28, 36] were further excluded with a final result of r = 0.33, (95% CI = 0.2 to 0.47, p < 0.001; I2 = 0%,*p* = 0.7) (Fig. 3f).

**Fig. 4:**
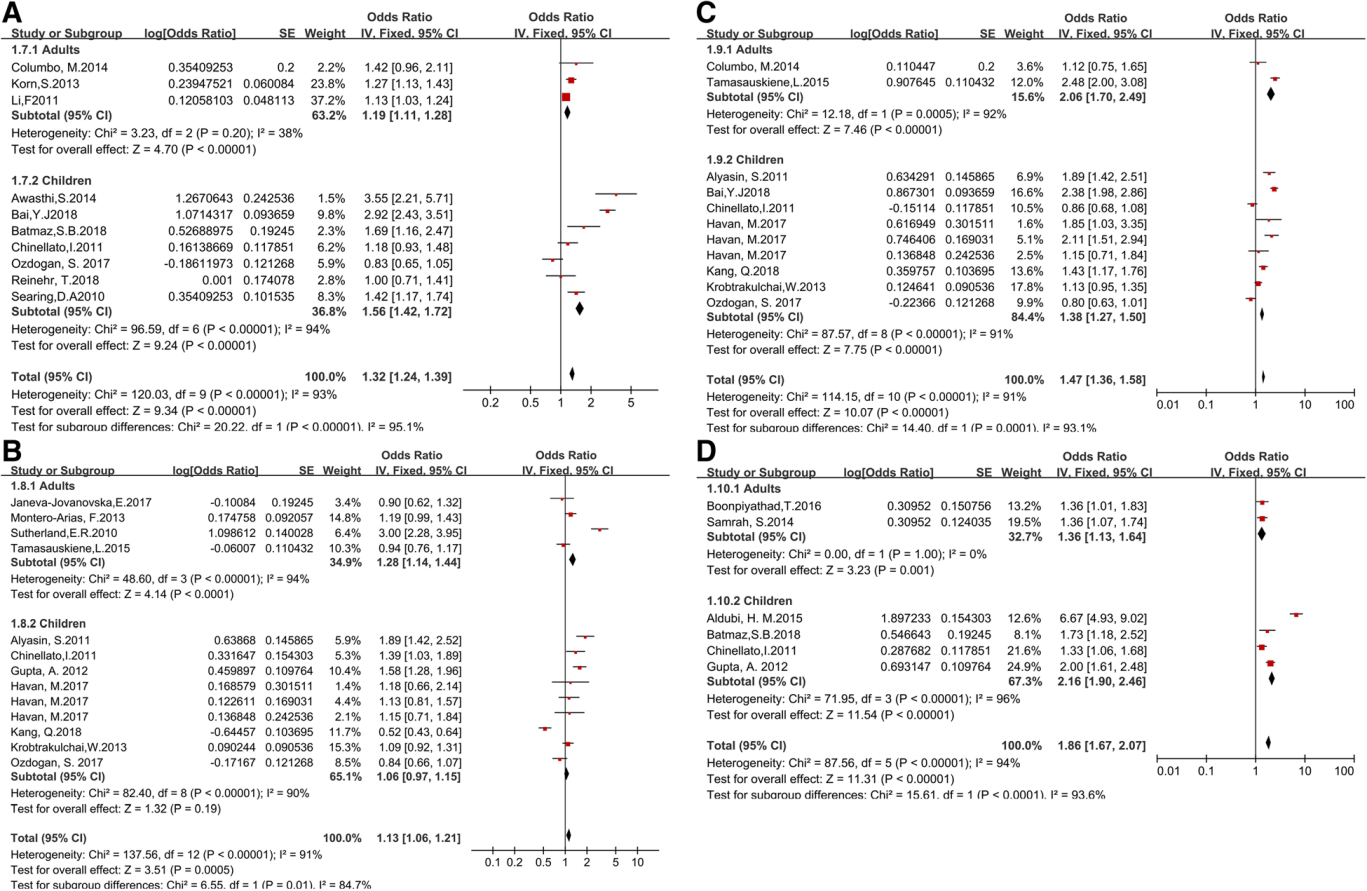
Forest plots of subgroup analysis of the relation between vitamin D and asthma lung function in two age groups: **a** FEV1%; **b** FEV1; **c** FEV1/FVC; **d** ACT

Lastly, we performed a subgroup analysis based on two age groups (children and adults). The negative correlation between vitamin D values and lung function in asthma patients remained significant in both age groups (Fig. 4a-d).

### Publication bias

Symmetrical funnel plots suggested no significant publication bias (Additional file 2: Figure S2-Figure S4).

## Discussion

This systematic review and meta-analysis investigated specifically the relation between vitamin D and lung function in both adults and children with asthma. We identified 27 studies with independent data and found that serum vitamin D was positively correlated with lung function in asthma patients, as determined by FEV1, FEV1%, FEV1/FVC, FVC%, FVC and ACT scores. Subgroups analysis revealed that this positive correlation exists irrespective of age. Furthermore, participants with insufficient or deficient vitamin D levels have slightly poorer lung function than those with sufficient levels. Inspection of the included studies revealed some heterogeneity in the duration of therapy, categorization threshold and population. Our research outcome confirmed the results reported by Zhang LL [9], in which vitamin D deficiency mirrored a remarkable decrease in lung function in asthmatic children. However, our evidence is much more robust, as we included more recent published studies.

vitamin D receptors (VDRs) are widely distributed in respiratory epithelial cells and immune cells (B cell, T cell, macrophages and monocytes) [27, 49], and the active form of vitamin D (1,25(OH)2D3) exerts its physiological effects by binding to VDRs. Binding of vitamin D the VDR strengthens the coactivation of VDR with the retinoid X receptor (RXR). Vitamin D combined with VDR-RXR heterodimers interact with vitamin D response elements (VDREs) to promote vitamin D-regulated gene transcription. The gene encoding 25-dihydroxy vitamin D3 24-hydroxylase, CYP24A, is the well-known 1,25(OH)2D3-responsive gene and has various VDREs in its promoter. CYP24A belongs to the cytochrome P450 (CYP) family. The P450 (CYP) family encodes multiple enzymes that are used in the oxidative metabolism of many endogenous and exogenous compounds [50, 51].

Considering the presence of VDRs on immune cells and various tissues in the airways, the role of vitamin D as a potentially modifiable factor in asthma has generated much interest regarding its purported immunomodulatory function. Serum 25(OH)D is used as a marker of vitamin D standards clinically [52]. The activating enzyme 1a-hydroxylase is expressed by respiratory epithelial cells, and converts inactive 25(OH)D to the active 1, 25(OH)2D form [53–55]. Through binding to VDRs, 1,25(OH)2D directly or indirectly interacts with various immune cells. VDRs regulate the transcription of diverse genes related to immunomodulation and inflammation [56]. In addition, vitamin D inhibits IL-4-mediated expression of IL-13 and the proinflammatory cytokines IL-17, and accelerates T-regulatory cell (Treg) secretion of anti-inflammatory cytokines, such as IL-10 [57, 58]. Additionally, 25(OH)2D may shift the balance of the T-regulatory lymphocyte reaction from the TH1 phenotype to the relatively less inflammatory TH2 phenotype [59, 60].

Our findings of the correlation between serum 25(OH)D concentrations and asthma lung function are supported by the current biological knowledge. In terms of the Impact on lung structural development: vitamin D deficiency could exacerbate lung structure and generate deficits in lung function, thus creating permanent susceptibility to poorer respiratory outcomes. In terms of airway smooth muscle (ASM) remodelling: ASM cells possess the enzymatic mechanisms to transform 25-hydroxy vitamin D to 1, 25-dihydroxy vitamin D, and 1, 25-dihydroxy vitamin D in turn inhibits ASM proliferation and suppresses expression of inflammatory chemokines [31, 61, 62]. The precise signalling mechanisms remain unclear, but may involve the phosphorylation of checkpoint kinase-1 and the diminished hyperphosphorylation of retinoblastoma protein induced by PDGF [63–65]. This process further leads to a decline in airflow and a decrease in small airway obstruction, which has direct relevance for lung function and airway remodelling in asthma patients [66]. Immunomodulation with vitamin D added to human monocytes may restrain the expression of Toll-like receptors 2 and 4, resulting in reduced tumour necrosis factor alpha (TNF-a) production. In terms of anti-inflammatory influence: vitamin D receptor (VDR) could suppress NF-kB activation and signaling, and vitamin D could inhibit the synthesis of pro-inflammatory cell factors. In terms of changing the outcome of anti-asthmatic therapy: vitamin D could enhance corticosteroid responsiveness through increasing mitogen-activated protein kinase 1 (MKP-1), which is a protein involved in directing cellular responses to a diverse array of stimuli. The VDR may cause downregulation of corticosteroid pathways. In terms of respiratory tract infections: vitamin D deficiency can induce infections by influencing the production of antimicrobial peptides. 1,25(OH)2D was found to be related to an antimicrobial peptide called cathelicidin [67, 68]. This peptide colocalises within phagosomes and is known to be active against a wide variety of mycobacteria, viruses, bacteria and fungi. Through these mechanisms, vitamin D deficiency can lead to a higher susceptibility to infection, poorer lung function and more severe asthma exacerbations.

When the correlation coefficient group was further subgrouped into two age groups, children (ages< 18 years) and adults (ages> 18 years), we found the reported associations were still significant in both subgroups. This finding differs from previous studies that reported a negative correlation only in the paediatric group [69–71]. A possible explanation may be that the serum vitamin D levels were the same in the adults and the children (21.5 ± 11.40 ng/ml and 21.11 ± 14.50 ng/ml, *p* > 0.05). Further studies are needed to determine whether serum IgE levels and serum vitamin D are inversely related in the two age groups, which could help to confirm that vitamin D supplementation may be beneficial in downregulating allergic responses in adults and children.

There was a positive trend in the relation between vitamin D and two indexes (FEV1/FVC and FVC%), but it did not reach significance. This finding may be due to the relatively small number of included studies and the high between-study heterogeneity.

Our study has several strengths. First, compared to previous related systematic reviews, we identified more observational studies (both case-control and cohort) in the analysis. Second, as a consequence of our more comprehensive approach to includ studies, along with data abstraction and meticulous risk of bias evaluation methodology with the risk of bias tool recommended by the Cochrane Collaboration [31, 62], we were able to identify the presence of heterogeneity between studies, which led to a more conservative conclusion. Third, the proportion of the selected participants with missing outcome data was small, and serum 25(OH)D levels were measured with validated assays in high quality laboratories. Fourth, as this meta-analysis was based on published literature, publication bias that results from a tendency to report only positive results is also a consideration. However, the symmetric funnel plot indicated that such bias was minimal.

Some limitations also existed in our studies. The power for some subgroup analyses was limited, and relatively large heterogeneity was noted; this problem is unavoidable, considering the small number of studies reporting data from adults in this field. Moreover, most of the studies evaluated only one measurement of circulating 25(OH)D levels, and the time for the blood sample collection was not always consistent. Furthermore, different therapeutic drugs can modulate serum vitamin D levels. Nevertheless, the results remained concordant in terms of the extracted correlation coefficient, and most of our final results exhibited no significant heterogeneity. Finally, we are unable to use these results to propose specific treatment strategies because of limited information.

## Conclusion

The pooled estimates from the observational studies show that high blood vitamin D levels can benefit lung function and slow asthma exacerbation. Due to the limited data, we are unable to determine an optimal cut-off dose of vitamin D for asthma lung function and control. More comprehensive randomised controlled clinical trials with sufficient power and longer follow-up duration are needed to confirm the results.

## Additional files

Additional file 1: PRISMA checklist

Additional file 2: Figure S1. Conversion formulas. Figure S2. Funnel plot for lung function in asthma patients with different vitamin d levels: A. FEV1; B. FEV1/FVC. Figure S3. Funnel plot of the relation between vitamin d and lung function and exacerbations in asthma patients: A. FEV1; B. FEV1%; C. FVC; D. FVC%; E. FEV1/FVC; F. ACT. Figure S4. Funnel plot of subgroup analyses of the relation between vitamin d and lung function and exacerbations in asthma patients: A. FEV1%; B. FEV1; C. FEV1/FVC; D. ACT

## Abbreviations

ACT: Asthma control test
ASM: Airway smooth muscle
FEV1: Forced expiratory volume in one second
FVC: Forced vital capacity
MOOSE: Meta-analysis of Observational Studies in Epidemiology
NOS: Newcastle–Ottawa Scale
PRISMA: Preferred Reporting Items for Systematic Reviews and Meta-Analyses
r: Correlation coefficient
RXR: Retinoid X receptor
SE: Standard errors
TH: T helper
TNF-a: Tumour necrosis factor alpha
USA: United States of America
VDREs: Vitamin D response elements
VDRs: Vitamin D receptors
